# Prognostic impact of MutT homolog‐1 expression on esophageal squamous cell carcinoma

**DOI:** 10.1002/cam4.979

**Published:** 2016-12-05

**Authors:** Shingo Akiyama, Hiroshi Saeki, Yuichiro Nakashima, Makoto Iimori, Hiroyuki Kitao, Eiji Oki, Yoshinao Oda, Yusaku Nakabeppu, Yoshihiro Kakeji, Yoshihiko Maehara

**Affiliations:** ^1^ Department of Surgery and ScienceGraduate School of Medical SciencesKyushu UniversityFukuokaJapan; ^2^ Division of Gastrointestinal SurgeryGraduate School of MedicineKobe UniversityKobeJapan; ^3^ Department of Molecular OncologyGraduate School of Medical Sciences, Kyushu UniversityFukuokaJapan; ^4^ Innovative Anticancer Strategy for Therapeutics and Diagnosis GroupInnovation Center for Medical Redox NavigationKyushu UniversityFukuokaJapan; ^5^ Department of Anatomic PathologyGraduate School of Medical SciencesKyushu UniversityFukuokaJapan; ^6^ Division of Neurofunctional GenomicsDepartment of Immunobiology and NeuroscienceMedical Institute of BioregulationKyushu University, FukuokaJapan

**Keywords:** Esophageal cancer, MutT homolog‐1, oxidative stress, oxidized nucleotides, prognosis

## Abstract

MutT homolog‐1 (MTH1) is a pyrophosphatase that acts on oxidized nucleotides and hydrolyzes 8‐oxo‐2’‐deoxyguanosine triphosphate in deoxynucleoside triphosphate pool to prevent its incorporation into nuclear and mitochondrial DNA, result in reduce cytotoxicity in tumor cells. MTH1 is overexpressed in various cancers and is considered as a therapeutic target. Environmental factors such as cigarette smoking and alcohol consumption are critical risk factors for the development and progression of esophageal squamous cell carcinoma (ESCC), suggesting that oxidative stress contributes to the pathogenesis of ESCC. We examined the expression of MTH1 and the accumulation of 8‐oxo‐2’‐deoxyguanosine (8‐oxo‐dG) in 84 patients with ESCC who underwent curative resection without neoadjuvant therapy. *MTH1* mRNA level was quantified by performing quantitative reverse transcription‐PCR. Immunohistochemical analysis of paraffin‐embedded cancer tissues was performed to determine MTH1 protein expression and 8‐oxo‐dG accumulation. *MTH1* mRNA expression was higher in cancerous tissues than in the corresponding normal epithelium (*P *< 0.0001). Immunohistochemical analysis showed that high MTH1 expression was significantly associated with deeper tumor invasion and venous invasion, advanced cancer stage, and poor overall survival (*P *= 0.0021) and disease‐specific survival (*P* = 0.0013) compared with low MTH1 expression. Furthermore, high MTH1 expression was an independent predictor of poor disease‐specific survival (*P *= 0.0121). In contrast, 8‐oxo‐dG accumulation was not associated with any clinicopathological factor and poor prognosis. These results suggest that MTH1 overexpression is a predictor of ESCC progression and poor prognosis and that MTH1 can serve as a therapeutic target for treating patients with ESCC.

## Introduction

Esophageal cancer is associated with poor prognosis despite improvements in treatment outcomes through advances in diagnostic and therapeutic strategies such as endoscopic resection, surgery, radiotherapy, and chemotherapy 1, 2, 3. Esophageal squamous cell carcinoma (ESCC) accounts for >90% cases of esophageal cancer in East Asia, including Japan, whereas esophageal adenocarcinoma accounts for >50% cases of esophageal cancer in Europe and the United States 4. Environmental factors such as cigarette smoking and alcohol consumption are suggested to contribute to the carcinogenesis of ESCC. The risk of ESCC increases by >50‐fold in individuals who smoke heavily and drink a lot of alcohol compared with that in individuals who do not smoke or drink alcohol 5. In addition, genetic factors are strongly associated with the carcinogenesis of ESCC 6. To improve treatment outcomes of patients with ESCC, it is necessary to determine the biological features of ESCC and to identify key factors associated with the prognosis of ESCC.

Cells are constantly exposed to reactive oxygen species (ROS). Levels of ROS in cells are determined by both their environment (i.e., hypoxia or exposure to cigarette smoke) and intrinsic characteristics (i.e., Warburg effect). ROS react with lipids, proteins, and nucleic acids and convert them into their oxidized forms 7. Moreover, ROS attack nucleotides present in deoxynucleoside triphosphate (dNTP) pool as well as within DNA and convert them into their oxidized forms. Particularly, the guanine base is the most susceptible to oxidation, and presence of its oxidized form, that is, 8‐oxo‐2’‐deoxyguanosine (8‐oxo‐dG), in DNA is an indicator of oxidative DNA damage 7, 8, 9. Increased 8‐oxo‐dG levels have been detected in the lung tissues 10 or ESCC samples from patients with a high smoking index 11. Presence of oxidized purine nucleosides in nuclear and mitochondrial DNA induces mutations during DNA replication or DNA strand breaks because of base excision repair, which may lead to cellular transformation, cellular senescence, or cell death and eventually various diseases. 12, 13.

MutT homolog‐1 (MTH1) is a pyrophosphatase that hydrolyzes oxidized purine dNTPs into their monophosphate forms and prevents their incorporation into nuclear and mitochondrial DNA 7, 14. Because MTH1 removes cytotoxic oxidized dNTPs, an association may exist between MTH1 expression and tumor progression 15. MTH1 expression is increased in various cancer cell lines 16 and in clinical specimens of lung cancer 17, renal carcinoma 18, brain tumors 19, and gastric cancer 20. Importantly, small‐molecule inhibitors of MTH1 exerts tumor‐specific cytotoxic effects, suggesting that it can be used as a candidate for developing a novel anticancer drug 16, 21.

In this study, we analyzed MTH1 expression in clinical specimens obtained from patients with ESCC to determine its clinical significance. Our results suggested that MTH1 expression was a biomarker of ESCC progression and poor prognosis.

## Materials and Methods

### Cell lines

The study included nine ESCC cell lines (TE1, TE2, TE3, TE5, TE8, TE10, TE12, TE13, and TE15) obtained from RBC (Ibaraki, Japan), three human fibroblast cell lines (MRC5, BJ, and WI‐38) purchased from ATCC (Nashville, TN), and one HeLa cell line obtained from JCRB (Osaka, Japan). The ESCC, fibroblast, and HeLa cell lines were cultured in RPMI‐1640, EMEM, and DMEM, respectively, supplemented with 10% FBS and penicillin/streptomycin at 37°C and in 5% CO_2_. All cell lines were authenticated by short tandem repeat analysis.

### Patients and preparation of specimens

Between October 1996 and June 2011, 481 patients with ESCC underwent esophageal resection at our institute. Out of the 481 cases, we analyzed 84 patients who underwent curative surgery without any preoperative therapies or distant metastasis. The cases that underwent any neoadjuvant chemotherapy (including salvage operation) were not included in this study. Specimens for immunohistochemical analysis were fixed in 10% formalin solution after resection, embedded in paraffin, and cut into 5‐*μ*m‐thick slices. Histological diagnosis was performed according to the 7th edition of the Union for International Cancer Control‐TNM Classification. For isolating total RNA, normal epithelia and cancerous regions of specimens from 47 patients with ESCC were resected and were frozen immediately. The study was approved by the Ethics Committee of Kyushu University (Number: 26‐92).

### Preparation of cell blocks

The cells were transfected with siRNAs against *MTH1* (si*MTH1*; 5ʹ‐CGACGACAGCUACUGGUUU‐3ʹ) 16, 21 and luciferase (si*GL2*; 5ʹ‐CGUACGCGGAAUACUUCGA‐3ʹ) 22 using RNAiMAX reagent (Invitrogen, Carlsbad, CA, USA). The transfected cells were detached and fixed in formalin for 1 h. The fixed cells were collected by centrifugation and using OCT compound and 100% ethanol, were dehydrated, and were embedded in paraffin.

### Quantitative reverse transcription‐PCR

For quantitative reverse transcription‐PCR (qRT‐PCR), total RNA was extracted from each cell line using RNeasy Mini Kit (Qiagen, Hilden, Germany) or from the clinical specimens of 47 patients with ESCC (normal epithelium and cancerous region) using Trizol (Thermo Fisher Scientific, Waltham, MA, USA). Complementary DNA was synthesized from 1 *μ*g total RNA using High‐Capacity cDNA Reverse Transcription Kit (Applied Biosystems, Foster City, CA, USA). Quantitative PCR was performed using QuantiFast SYBR Green PCR Kit (Qiagen) and LightCycler 480 II (Roche Diagnostics, Basel, Switzerland). Relative expression of *MTH1* mRNA was normalized to that of *β‐actin* mRNA. Primer sequences used for performing the qPCR are as follows: *MTH1* #1 16 forward 5ʹ‐GTGCAGAACCCAGGGACCAT‐3ʹ and reverse 5ʹ‐GCCCACGAACTCAAACACGA‐3ʹ, *MTH1* #2 21 forward 5ʹ‐CTCAGCGAGTTCTCCTGG‐3ʹ and reverse 5ʹ‐GGAGTGGAAACCAGTAGCTGTC‐3ʹ, and *β‐actin* 23 forward 5ʹ‐GAAAATCTGGCACCACACCT‐3ʹ and reverse 5ʹ‐TAGCACAGCCTGGATAGCAA‐3ʹ.

### Western blotting

Cell pellets were lysed in RIPA buffer (1.0% NP‐40, 50 mmol/L Tris‐HCl [pH 8.0], 150 mmol/L NaCl, 0.5% deoxycholate, 0.1% SDS, and 1 mmol/L phenylmethylsulfonyl fluoride) containing 1× protease inhibitor cocktail and 1× phosphatase inhibitor cocktail (Nacalai Tesque, Kyoto, Japan), and was denatured by adding 1× SDS sample buffer (62.5 mmol/L Tris‐HCl [pH 6.8], 2.5% SDS, 0.002% bromophenol blue, 5% *β*‐mercaptoethanol, and 10% glycerol). Protein concentration was determined by performing Bradford XL assay (Bio‐Rad, Hercules, CA, USA), and samples containing equal amount of proteins (10 *μ*g/lane) were resolved by performing SDS‐PAGE. Protein bands were detected using ImageQuant LAS 4000 Mini (GE Healthcare, Chicago, Il, USA), and imaging data were quantified using ImageJ software (NIH). The following antibodies were used for western blotting: anti‐MTH1 rabbit polyclonal antibody, which was prepared as described previously 19, 24, and anti‐*β*‐actin antibody (AC‐74; Sigma‐Aldrich, St. Louis, MO, USA).

### Immunohistochemical analysis

Formalin‐fixed paraffin‐embedded specimens were deparaffinized with xylene and were rehydrated in ethanol. For the immunohistochemical analysis of MTH1, the specimens were treated with 3% H_2_O_2_ in methanol for blocking endogenous peroxidase activity and with 10% normal goat serum for blocking nonspecific reaction. Next, the specimens were incubated overnight with a primary antibody against MTH1 24 (dilution, 1:100) at 4°C. For the immunohistochemical analysis of 8‐oxo‐dG, the specimens were autoclaved for 10 min in citrate buffer (pH 6.0) after deparaffinization and rehydration to aid antigen retrieval. Further, the specimens were treated with 10% normal goat serum for blocking nonspecific reaction and were incubated overnight with a primary antibody against 8‐oxo‐dG (N45.1; dilution, 1:20; Japanese Aging Control Institute, Shizuoka, Japan) at 4°C. Next, the specimens were treated with 3% H_2_O_2_ in methanol for blocking endogenous peroxidase activity. Finally, the specimens were treated with secondary antibody (Dako EnVision/HRP Universal kit; Agilent Technologies, Santa Clara, CA, USA), were incubated with DAB, and were counterstained with Mayer's hematoxylin.

MTH1 immunoreactivity was graded by determining the proportion and intensity of immunostaining in the cancerous region of the specimens. Staining intensity was rated on a scale of 0–3 (0, no staining; 1, weak staining; 2, moderate staining; and 3, strong staining). The percentage of positively stained cells was scored on a scale of 0–4 (0, 0% positively stained cells; 1, 1%–25% positively stained cells; 2, 26%–50% positively stained cells; 3, 51%–75% positively stained cells; and 4, >75% positively stained cells). The final score was obtained by multiplying the score of staining intensity with that of the percentage of positively stained cells, with scores in the range of 0–7 indicating low expression and those in the range of 8–12 indicating high expression. Further, 8‐oxo‐dG immunoreactivity was graded based on the percentage of positively stained cells, that is, 0–50% positively stained cells indicating low expression and >51% positively stained cells indicating high expression. These immunoreactivities were evaluated microscopically by two independent pathologists who were blinded to the study protocol. Representative pictures were taken by NanoZoomer‐SQ digital slide scanner and NDP view software (Hamamatsu photonics K. K., Shizuoka, Japan).

### Statistical analysis

**Figure 1:**
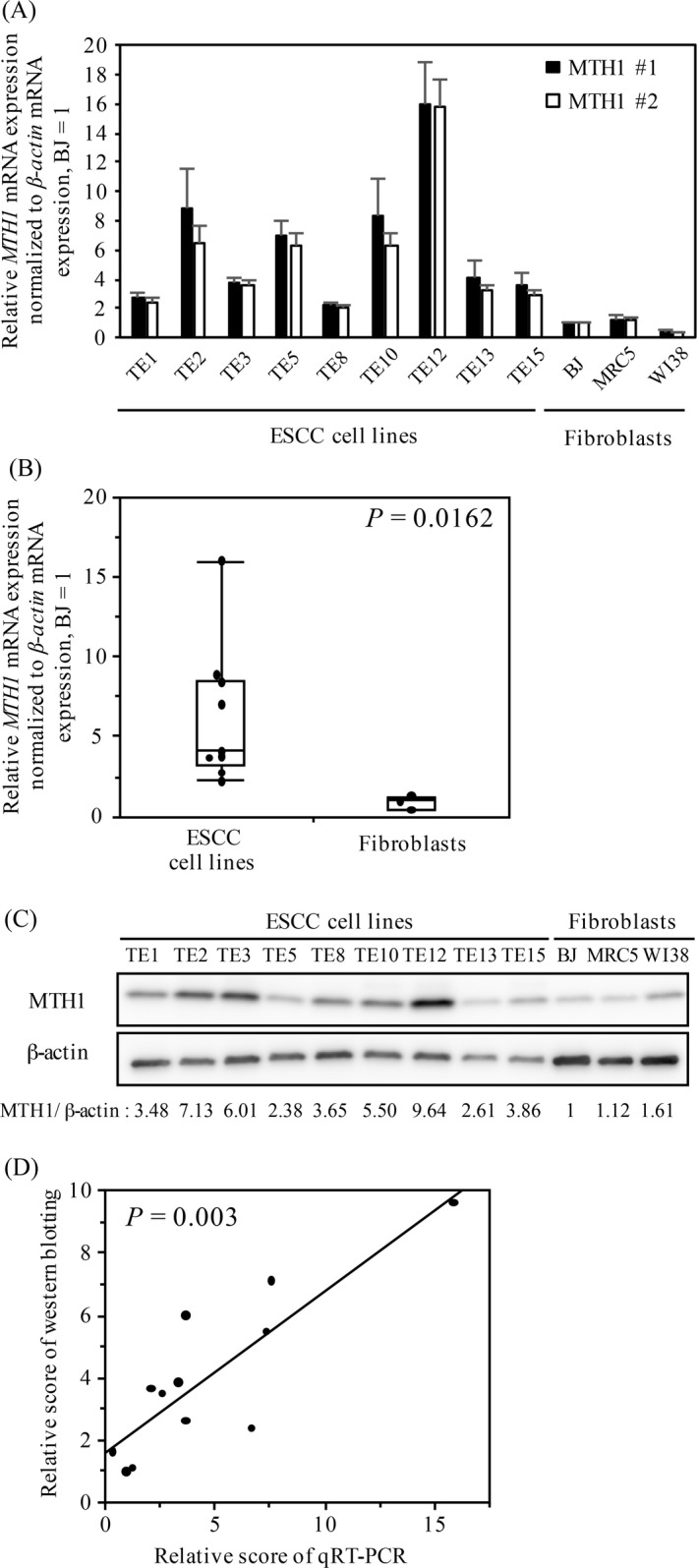
MTH1 expression in the ESCC and fibroblast cell lines. (A) *MTH1* mRNA expression in the ESCC and fibroblast cell lines. The mRNA levels were analyzed by performing qRT‐PCR. (B) Comparison of *MTH1* mRNA expression between the ESCC and fibroblast cell lines. The middle lines inside the boxes represent medians. The upper and lower box boundaries represent the 25th and 75th percentiles, respectively. The lower and upper whiskers extend to the lowest and highest values, respectively. *P* value was calculated using Mann–Whitney U test. (C) Western blotting of MTH1 in the ESCC and fibroblast cell lines. The scores above the images are for each lane in western blotting. (D) Correlation between MTH1 mRNA and protein expression in each cell line was determined using Spearman's rank correlation coefficient. ESCC, esophageal squamous cell carcinoma; qRT‐PCR, quantitative reverse transcription‐PCR.

**Figure 2:**
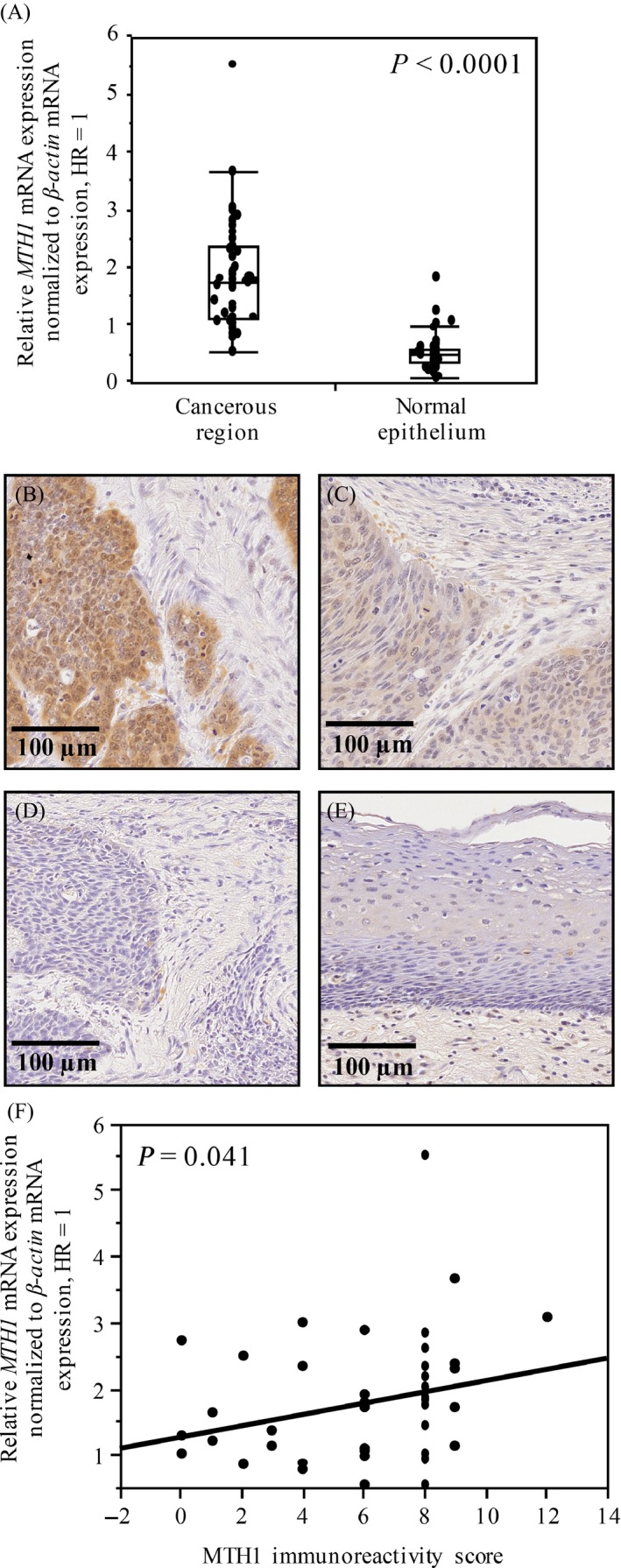
MTH1 expression in ESCC tissue samples. (A) Comparison of *MTH1* mRNA expression between normal epithelia and cancerous regions in ESCC tissue samples obtained from patients with ESCC. *P* value was calculated using Mann–Whitney *U* test. (B–E) Representative images of immunohistochemical analysis of MTH1 in ESCC tissue samples. (B) High MTH1 expression. (C and D) Low MTH1 expression. (C) Weak and focal MTH1 immunoreactivity. (D) Comparable MTH1 staining in the surrounding matrix. (E) MTH1 expression in the normal epithelium in (B). (F) Correlation between *MTH1* mRNA expression level and MTH1 immunoreactivity score in each case was determined using Spearman's rank correlation coefficient. ESCC, esophageal squamous cell carcinoma; qRT‐PCR, quantitative reverse transcription‐PCR.

Statistical analysis was performed using JMP software version 11 (SAS Institute, Cary, NC , USA). Spearman's rank correlation coefficient was used for measuring statistical dependence between two variables (Fig. 1A, D, and 2F). Mann–Whitney U test was used when comparing means from two divided groups (Fig. 1B and 2A). Correlation between MTH1 expression and clinicopathological characteristics of patients with ESCC was analyzed using Fisher's exact test and Mann–Whitney U test. Kaplan–Meier methods were used to calculate overall survival (OS) and disease‐specific survival (DSS) rates, and log‐rank tests were used to compare differences between two groups. Multivariate analysis was performed using Cox regression model, and variables with probability (*P*) values of <0.05 were used in the final model. In all analyses, *P* < 0.05 was considered statistically significant.

## **Results**

### MTH1 expression in ESCC and fibroblast cell lines

MTH1 expression is higher in transformed cell lines than in nontransformed cell lines 16. To investigate this, we evaluated *MTH1* mRNA expression in the nine ESCC cell lines (TE1, TE2, TE3, TE5, TE8, TE10, TE12, TE13, and TE15) and three human fibroblast cell lines (MRC5, BJ, and WI‐38) by performing qRT‐PCR with two independent primer sets that specifically amplified *MTH1* coding region (Fig. 1A). Scores of qRT‐PCR obtained using the two independent primer sets were significantly correlated (*P *< 0.0001). Although *MTH1* mRNA expression level varied among the nine ESCC cell lines, the average *MTH1* mRNA expression level was significantly higher in the ESCC cell lines than in the fibroblast cell lines (*P *= 0.0162; Fig. 1B).

Next, we performed western blotting to evaluate MTH1 protein expression level in these cell lines (Fig. 1C). Normalized MTH1 protein expression level also varied among the nine ESCC cell lines. However, MTH1 protein expression level was significantly higher in the ESCC cell lines than in the fibroblast cell lines (*P *= 0.0162). In addition, scores of MTH1 protein expression were significantly correlated with those of *MTH1* mRNA expression (*P *= 0.003; Fig. 1D). These results indicated that MTH1 mRNA and protein expression levels in the ESCC cell lines were significantly higher than those in nontransformed fibroblast cells and that MTH1 protein expression was mainly controlled at the transcription level.

### MTH1 expression in ESCC tissue samples

Next, we evaluated MTH1 expression in specimens obtained from patients with ESCC. First, we examined *MTH1* mRNA expression in matched pairs of cancerous region and normal epithelium in specimens obtained from available 47 patients with ESCC. *MTH1* mRNA expression level in the cancerous region was significantly higher than that in the normal epithelium (*P *< 0.0001; Fig. 2A).

Next, we examined MTH1 protein expression in the paraffin‐embedded specimens obtained from patients with ESCC by performing immunohistochemical analysis. To confirm that the anti‐MTH1 antibody specifically detected MTH1 even in paraffin‐embedded tissues, we prepared paraffin blocks of HeLa cells treated with si*MTH1* or si*GL2* (control) and immunostained these blocks using the anti‐MTH1 antibody. Efficiency of si*MTH1* was confirmed by performing western blotting (Fig. S1A). Strong intracellular immunoreactivity was observed in cell blocks containing si*GL2*‐treated cells (Fig. S1B), whereas only a weak signal was observed in cell blocks containing si*MTH1*‐treated cells (Fig. S1C), confirming the specific reactivity of the anti‐MTH1 antibody in paraffin‐embedded specimens.

Next, we immunostained paraffin‐embedded tumor specimens of 84 patients with ESCC using the anti‐MTH1 antibody. In most specimens, MTH1 immunoreactivity in the normal epithelium (Fig. 2E) and surrounding stromal regions (Fig. 2B–D) was relatively weaker than that in the cancerous region. The scores of MTH1 immunoreactivity were significantly correlated with those of MTH1 mRNA expression in ESCC tissue samples (*P* = 0.041; Fig. 2F). In all, 32 (38%) patients whose tumor specimens showed strong and diffuse MTH1 immunoreactivity were categorized in the high MTH1 expression group (Fig. 2B) and 52 (62%) patients whose tumors showed weak and focal MTH1 immunoreactivity (Fig. 2C) or immunoreactivity comparable to that of the surrounding matrix (Fig. 2D) were categorized in the low MTH1 expression group. High MTH1 expression was significantly associated with pT3,4 (*P *= 0.0008), venous invasion (*P *= 0.0463), and pStage III (*P *= 0.0434) but was not significantly associated with other factors (Table 1).

**Table 1:** Correlation between clinicopathological characteristics and MTH1 expression

Factor	Low MTH1 expression (*n* = 52)	High MTH1 expression (*n* = 32)	*P* value
Age (years)			
Median ± SD	63.5 ± 8.1	64.0 ± 10.4	0.787
Gender			
Male	47 (90.4)	26 (81.3)	0.319
Female	5 (9.6)	6 (18.7)	
Tumor differentiation			
Well to moderate	44 (84.6)	25 (78.1)	0.560
Poor	8 (15.4)	7 (21.9)	
Tumor depth			
pT1, 2	33 (63.5)	8 (25.0)	0.0008
pT3, 4	19 (36.5)	24 (75.0)	
Lymph node metastasis			
(‐)	22 (42.3)	12 (37.5)	0.819
(+)	30 (57.7)	20 (62.5)	
Lymphatic invasion			
(‐)	24 (46.1)	13 (40.6)	0.657
(+)	28 (53.9)	19 (59.4)	
Venous invasion			
(‐)	33 (63.5)	13 (40.6)	0.046
(+)	19 (36.5)	19 (59.4)	
Stage			
pStage I, II	32 (61.5)	12 (37.5)	0.043
pStage III	20 (38.5)	20 (62.5)	
*P53* mutation (*n* = 60)			
(‐)	13 (36.1)	8 (33.3)	1
(+)	23 (63.9)	16 (66.7)	

### Expression of MTH1 and prognosis of patients with ESCC

**Figure 3:**
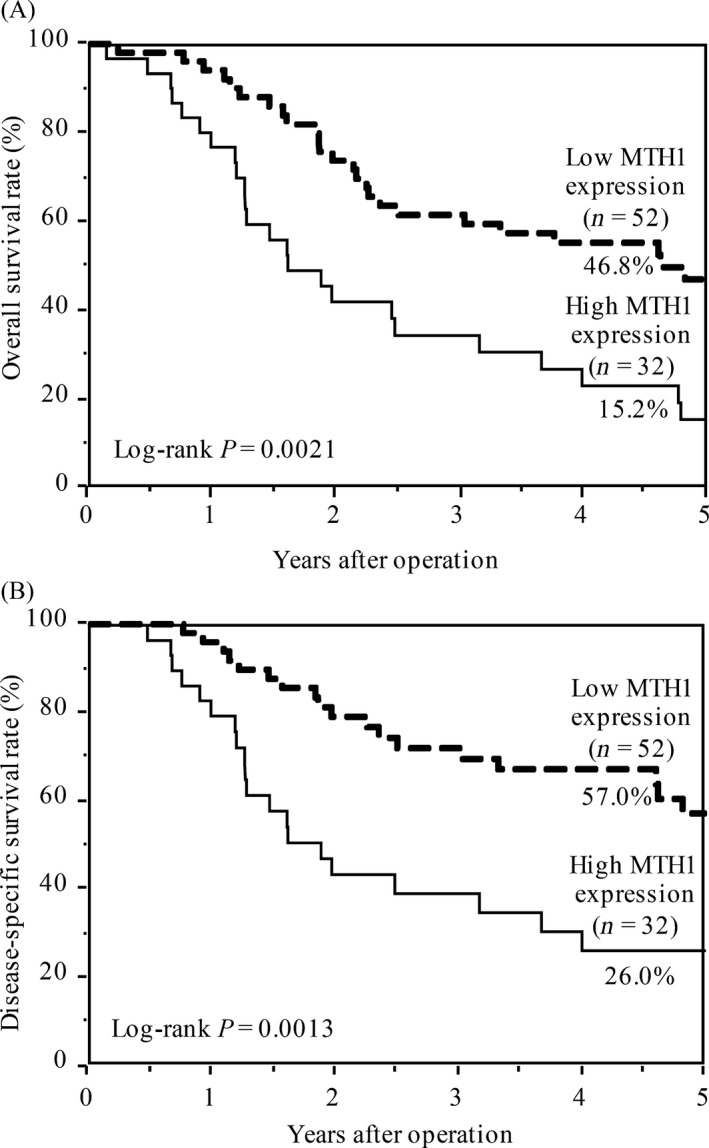
Kaplan–Meier curves for (A) overall survival rate and (B) disease‐specific survival rate of patients with ESCC according to MTH1 expression. *P* value was calculated using the log‐rank test. ESCC, esophageal squamous cell carcinoma.

MTH1 overexpression in cancer cells promotes their proliferation by removing cytotoxic oxidized nucleotides 16. In this study, patients in the high MTH1 expression group showed significantly poorer OS (*P* = 0.0021; Fig. 3A) and DSS (*P *= 0.0013; Fig. 3B) than patients in the low MTH1 expression group. Univariate analysis indicated that tumor depth, lymph node metastasis, lymphatic invasion, venous invasion, and MTH1 expression were correlated with poor DSS rate (Table 2). Furthermore, multivariate analysis using the Cox regression model indicated that MTH1 expression was an independent predictor of poor DSS (Table 2).

**Table 2:** Univariate and multivariate analyses of the predictors of disease‐specific survival

Factor	Examination object	Comparable object	Univariate analysis			Multivariate analysis		
			HR^a^	95% CI^b^	*P* value	HR^a^	95% CI^b^	*P* value
Age (years)	≥65	<65	0.855	0.452–1.612	0.6268			
Gender	Male	Female	0.509	0.226–1.360	0.1634			
Tumor differentiation	Poor	Well to Moderate	2.032	0.963–4.003	0.0619			
Tumor depth	pT3/T4	pT1/T2	3.540	1.836–7.167	0.0001	1.903	0.911–4.135	0.0874
Lymph node metastasis	pN (+)	pN (−)	3.288	1.613–7.412	0.0008	1.681	0.741–4.167	0.2211
Lymphatic invasion	Ly (+)	Ly (−)	2.763	1.422–5.696	0.0024	1.772	0.813–4.096	0.1531
Venous invasion	V (+)	V (−)	3.250	1.691–6.432	0.0004	1.761	0.848–3.752	0.1297
MTH1 expression	High	Low	2.710	1.435–5.136	0.0023	2.368	1.211–4.641	0.0121

a HR, Hazard ratio.

b CI, confidence interval.

### 8‐oxo‐dG accumulation in ESCC tissue samples

8‐oxo‐dG immunoreactivity is an indicator of the accumulation of oxidized nucleosides in DNA 25. To evaluate the role of MTH1 in the removal of these oxidized nucleotides, we assessed 8‐oxo‐dG accumulation in specimens obtained from patients with ESCC by performing immunohistochemical analysis. In all, 43 (51%) patients showed high 8‐oxo‐dG accumulation (Fig. S2A) and 41 (49%) patients showed low 8‐oxo‐dG accumulation (Fig. S2B). Further, 8‐oxo‐dG accumulation was not associated with any clinicopathological factor (Table S1) and poor prognosis (Fig S3). In addition, no correlation was observed between MTH1 expression and 8‐oxo‐dG accumulation (Table S1).

## Discussion

ESCC is one of the most devastating cancers. Because environmental factors such as cigarette smoking or alcohol consumption are critical risk factors for the carcinogenesis and progression of ESCC, a strong correlation may exist between ESCC malignancy and oxidative stress. MTH1 is a pyrophosphatase of oxidized purine dNTPs, which exert cytotoxic effects on tumor cells by incorporating into DNA and by inducing DNA strand breaks. In this study, we found that MTH1 expression increased in the ESCC cell lines (Fig. 1) and in the cancerous regions of ESCC tissue samples (Fig. 2). Furthermore, patients with ESCC showing strong and diffuse MTH1 immunoreactivity had higher cancer stage (Table 1) and showed poorer prognosis than those showing weak and focal MTH1 immunoreactivity (Fig. 3). Importantly, high MTH1 expression was an independent predictor of poor DSS (Table 2). Since, this cohort only included cases that had not undergone preoperative therapy, our data indicate that MTH1 expression is a prognostic factor for patients who did not receive preoperative therapy. On the other hand, since little clinical data are available regarding postoperative therapy, the relationship between MTH1 expression and the effect of postoperative therapy could not be evaluated. We need to study this aspect in the future. To the best of our knowledge, this is the first study to show a correlation between MTH1, which is involved in the removal of oxidized nucleotides, and malignancy of ESCC.

Advanced‐stage renal cell carcinomas show significantly higher *MTH1* mRNA expression than early stage renal cell carcinomas 18. In patients with non‐small‐cell lung carcinomas, *MTH1* mRNA overexpression is significantly correlated with tumor pathological stage, lymph node metastasis, and poor prognosis 26. These evidence suggest that MTH1 expression is correlated with advanced cancer stage, tumor invasiveness, and poor prognosis. Because MTH1 is a pyrophosphatase of oxidized purine dNTPs, these data suggest that removal of oxidized dNTPs from the dNTP pool contributes to tumor progression or maintenance of malignant features. Cellular ROS levels increase during tumor progression because tumors rely on aerobic glycolytic pathway (also known as Warburg effect) for energy production. Environmental factors such as heavy smoking increase cellular ROS levels. High MTH1 expression may remove oxidized dNTPs and prevent their incorporation into DNA, thus promoting tumor survival and proliferation and contributing to the poor prognosis of patients with ESCC. In contrast, 8‐oxo‐dG accumulation in our cohort was not correlated with any clinicopathological factor (Table S1) or with poor prognosis (Fig. S3). 8‐oxo‐dG accumulation in the tumor is determined by multiple factors: 1 ROS‐induced production of 8‐oxo‐dG or 8‐oxo‐dGTP, 2 sanitization of 8‐oxo‐dGTP by MTH1, and 3 OGG1‐mediated excision of 8‐oxo‐dG from genomic DNA. Our data indicate that the potential of MTH1 to sanitize 8‐oxo‐dGTP in the nucleotide pool, rather than 8‐oxo‐dG accumulation itself, is a better predictor of prognosis in patients with ESCC. Other studies indicated that aberrant stabilization of NRF2, a master transcriptional regulator that integrates antioxidant response 27, was also significantly associated with the poor prognosis of patients with ESCC 28, indicating that suppression of cellular ROS production promoted tumor progression. The clinical impact of MTH1 on poor prognosis observed in this study suggests that, of the various factors required for coping with cellular ROS, removal of oxidized dNTPs plays a predominant role in the progression and survival of advanced ESCC. This hypothesis should be confirmed by analyzing other cohorts or by performing in vitro studies.

MTH1 expression was specifically increased in the cancerous regions of ESCC tissue samples (Fig. 2). In non‐small‐cell lung carcinoma, *MTH1* mRNA expression level is correlated with KRAS mutation and expression level 29. MTH1 expression may affect the optimal development and progression of RAS‐driven tumors 15. However, the frequency of KRAS mutations is extremely low in ESCC 30. The MTH1 promoter contains a consensus sequence for the binding of Ets family transcription factors, including NF‐*κ*B and AP‐1 31, whose activity is correlated with inflammation status. Previous studies indicate that NF‐*κ*B is constitutively active in ESCC 32 and that Ets‐1 expression is correlated with the invasion of ESCC 33, 34. ESCC incidence and progression are strongly correlated with cigarette smoking or alcohol consumption 5. Exposure of the esophageal duct to such environmental factors may induce inflammation. This inflammation of the esophageal epithelium might activate NF‐*κ*B and Ets family transcription factors, thus enhancing MTH1 expression.

Our findings indicate that MTH1 expression was altered during ESCC carcinogenesis and progression and suggest that MTH1 could be used as a predictive biomarker of poor prognosis in patients with ESCC. Our results also suggest that effective perioperative treatment should be considered for treating patients with ESCC who show high MTH1 expression. The potential of MTH1 inhibition as an anticancer therapy has been proposed and is currently under debate 16, 21, 35. Targeted therapy against MTH1 may be an ideal strategy for treating patients with advanced ESCC who show high MTH1 expression in their tumors and poor prognosis.

## Conflict of Interest

The authors declare no conflict of interest.

## Supporting information


**Figure S1.** MTH1 expression in siRNA‐transfected HeLa cells.

**Figure S2.** Accumulation of 8‐oxo‐dG in ESCC tissue samples.

**Figure S3.** Kaplan–Meier curves for overall survival rate and disease‐specific survival rate based on 8‐oxo‐dG accumulation.

**Table S1.** Correlation between clinicopathological characteristics and 8‐oxo‐dG accumulation.
