# Supplementary figures and images for: Prognostic impact of MutT homolog‐1 expression on esophageal squamous cell carcinoma
Source: Cancer Med. 2016 Dec 5;6(1):258–66. doi: 10.1002/cam4.979 (PMC5269568; doi:10.1002/cam4.979)

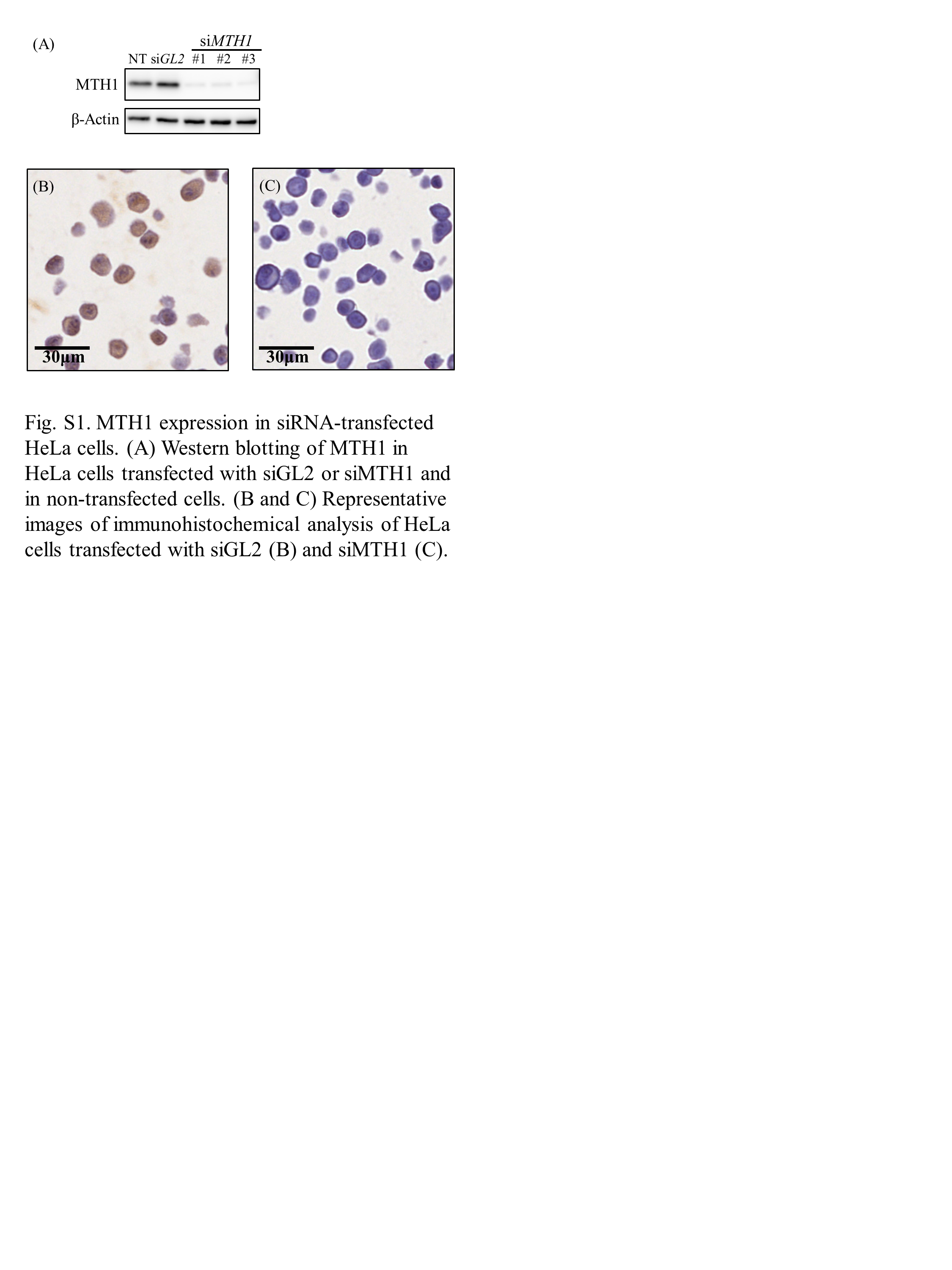

Supplement: Supplementary file 1 — Figure S1. MTH1 expression in siRNA‐transfected HeLa cells. [file CAM4-6-258-s001.tif]

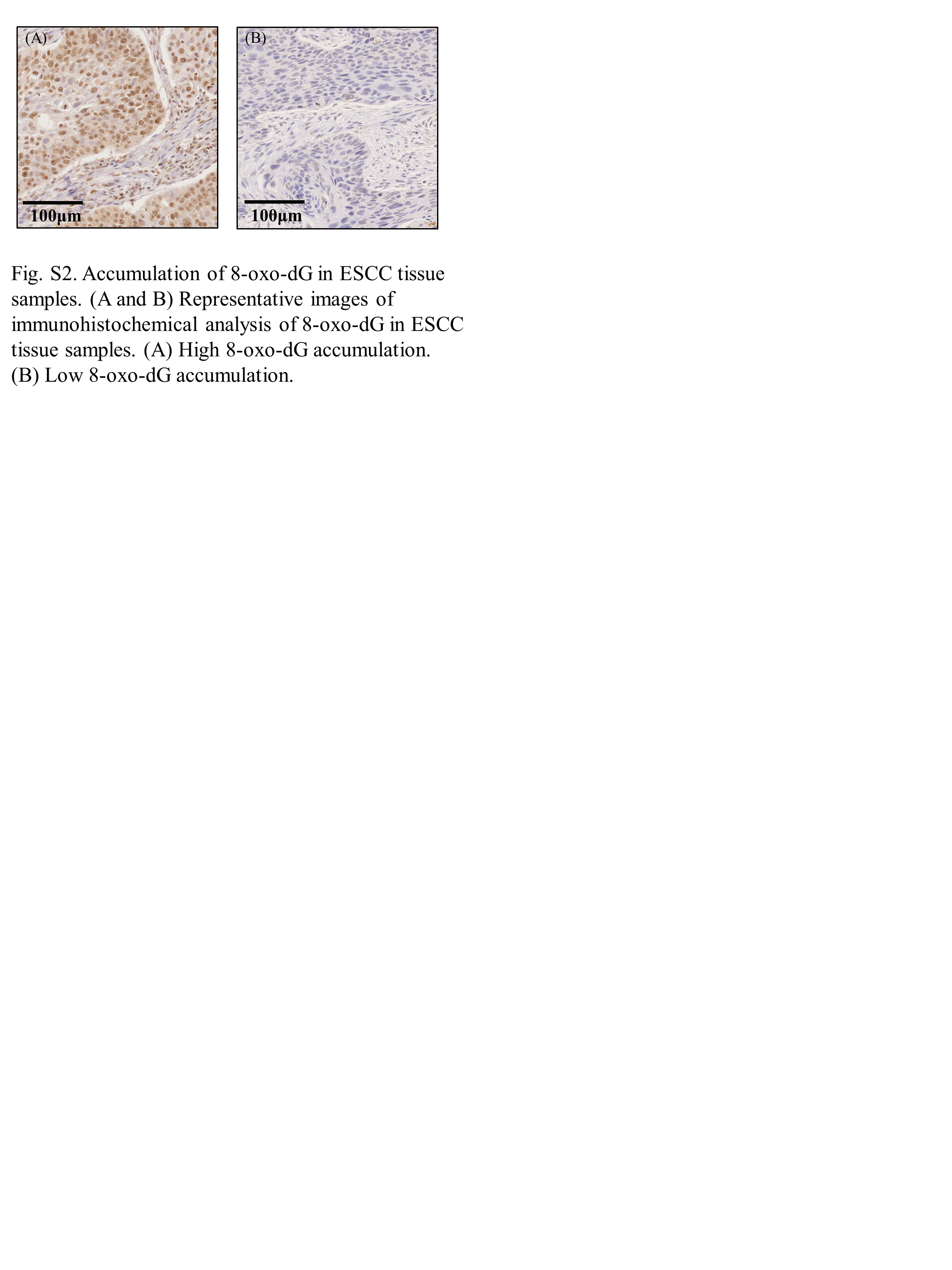

Supplement: Supplementary file 2 — Figure S2. Accumulation of 8‐oxo‐dG in ESCC tissue samples. [file CAM4-6-258-s002.tif]

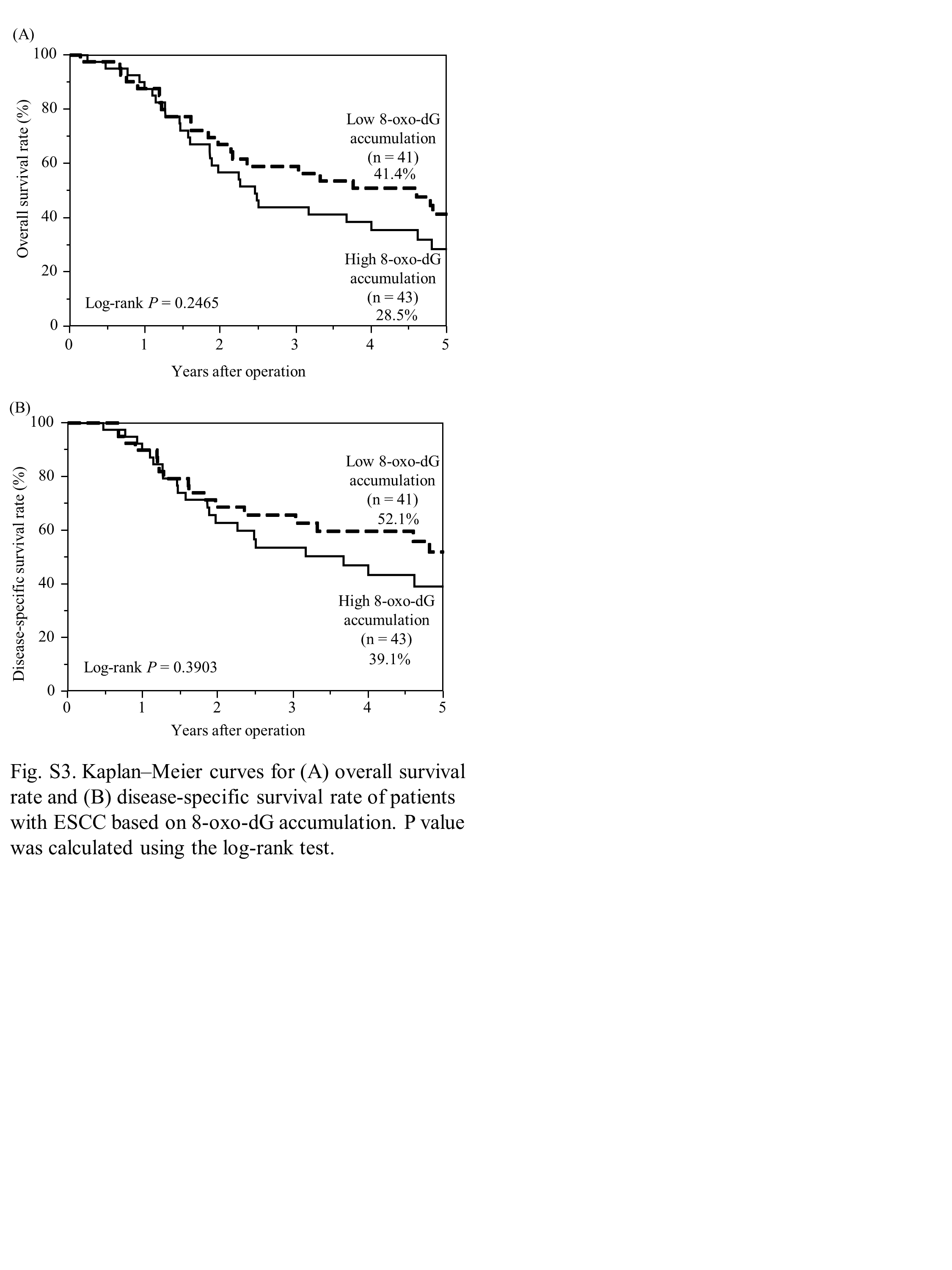

Supplement: Supplementary file 3 — Figure S3. Kaplan–Meier curves for overall survival rate and disease‐specific survival rate based on 8‐oxo‐dG accumulation. [file CAM4-6-258-s003.tif]
