# Supplementary material for: Prognostic impact of MutT homolog‐1 expression on esophageal squamous cell carcinoma
Source: Cancer Med. 2016 Dec 5;6(1):258–66. doi: 10.1002/cam4.979 (PMC5269568; doi:10.1002/cam4.979)
Supplement: Supplementary file 4 — Table S1. Correlation between clinicopathological characteristics and 8‐oxo‐dG accumulation. [file CAM4-6-258-s004.docx]

Table S1. Correlation between clinicopathological characteristics and 8-OHdG accumulation.

| Factor | Low 8-oxo-dG accumulation  (n = 41) | | High 8-oxo-dG accumulation  (n = 43) | | P value |
| --- | --- | --- | --- | --- | --- |
| Age (years) |  |  |  |  |  |
| median ± SD | 63.5 | ± 8.5 | 63.9 | ± 9.5 | 0.823 |
| Gender |  |  |  |  |  |
| Male | 35 | (85.4) | 38 | (88.4) | 0.754 |
| Female | 6 | (14.6) | 5 | (11.6) |  |
| Tumor differentiation |  |  |  |  |  |
| Well to moderate | 36 | (87.8) | 33 | (76.7) | 0.560 |
| Poor | 5 | (12.2) | 10 | (23.3) |  |
| Tumor depth |  |  |  |  |  |
| pT1, 2 | 17 | (41.5) | 24 | (55.8) | 0.200 |
| pT3, 4 | 24 | (58.5) | 19 | (44.2) |  |
| Lymph node metastasis |  |  |  |  |  |
| (-) | 16 | (39.0) | 18 | (41.9) | 0.827 |
| (+) | 25 | (61.0) | 25 | (58.1) |  |
| Lymphatic invasion |  |  |  |  |  |
| (-) | 14 | (34.1) | 23 | (53.5) | 0.083 |
| (+) | 27 | (65.9) | 20 | (46.5) |  |
| Venous invasion |  |  |  |  |  |
| (-) | 21 | (51.22) | 25 | (58.14) | 0.661 |
| (+) | 20 | (48.78) | 18 | (41.86) |  |
| Stage |  |  |  |  |  |
| pStage I, II | 19 | (46.3) | 25 | (58.1) | 0.382 |
| pStage III | 22 | (53.7) | 18 | (41.9) |  |
| P53 mutation (60) |  |  |  |  |  |
| (-) | 9 | (28.1) | 12 | (42.9) | 0.284 |
| (+) | 23 | (71.9) | 16 | (57.1) |  |
| MTH1 expression |  |  |  |  |  |
| MTH1 low | 24 | (58.5) | 28 | (65.1) | 0.654 |
| MTH1 high | 17 | (41.5) | 15 | (34.9) |  |
